# Complete Response With Trametinib in Advanced Low-Grade Serous Ovarian Carcinoma: A Case Report

**DOI:** 10.7759/cureus.53600

**Published:** 2024-02-05

**Authors:** Pedro Antunes Meireles, Beatriz Mira, Fátima Vaz

**Affiliations:** 1 Department of Medical Oncology, Instituto Português de Oncologia de Lisboa Francisco Gentil, Lisbon, PRT

**Keywords:** trametinib, molecular targeted therapy, mek inhibitor, ovarian cancer, case report

## Abstract

Low-grade serous ovarian carcinoma (LGSOC) is an uncommon subtype of ovarian cancer, and it is usually associated with reduced sensitivity to chemotherapy and worse outcomes. We present a case involving a 45-year-old female patient diagnosed with stage III-C low-grade serous ovarian carcinoma (LGSOC) in 2013. She achieved a complete response for 29 months after undergoing platinum-based chemotherapy and interval cytoreduction. However, in 2016, both local and distant relapses were observed. As there was no benefit from hormonal therapy and the patient refused chemotherapy, bevacizumab was initiated, resulting in disease stabilization for 30 months. At disease progression, trametinib was proposed, and the patient experienced an ongoing sustained complete response for over 36 months. To the best of our knowledge, this is the first report, outside of a clinical trial, regarding a complete response with single agent MEK inhibitor therapy in a patient with recurrent LGSOC, with unknown BRAF V600E mutation. We present the following case in accordance with the CAse REports (CARE) checklist.

## Introduction

Ovarian cancer is the eighth most prevalent cancer among females worldwide, and it represents the seventh most frequent cancer-related cause of mortality [1]. Up to 90% of ovarian cancers are of epithelial origin, with serous tumors being the most frequently diagnosed. Among these, low-grade serous ovarian carcinoma (LGSOC) is the least common subtype, affecting less than 5% of all patients with epithelial ovarian cancer [2]. The pathological diagnosis of LGSOC is primarily based on the assessment of nuclear atypia and the mitotic rate [3]. In contrast to high-grade serous carcinomas, low-grade disease has a mild-to-moderate nuclear atypia and lower mitotic index. There is biological evidence suggesting that LGSOC develops from a different pathway from high-grade serous ovarian carcinoma (HGSOC), having a high frequency of activating mutations in the mitogen-activated protein kinases (MAPK) pathway and generally expressing wild-type TP53 [4]. In the largest genetic study of LGSOCs to date, targeted sequencing identified 47% of cases with mutations in key RAS/RAF pathway genes (KRAS, BRAF, and NRAS), as opposed to the high-grade subtype, where BRAF mutations are scarce [5]. Furthermore, in the same cohort, the frequency of hormonal expression was 97% and 68% for estrogen and progesterone receptors, respectively. These observations may explain the potential efficacy of target therapy in LGSOC [6].

Data from a phase 3 trial suggests that the MAPK pathway activity is important, even in the absence of an identified canonical mutation, since trametinib, a MEK inhibitor, was effective in prolonging progression-free survival in advanced LGSOC, regardless of somatic KRAS, BRAF, or NRAS mutation [7].

Clinically, LGSOC is characterized by a younger age at diagnosis and less sensitivity to platinum-based chemotherapy [8]. Similarly, to high-grade serous carcinoma, most patients with low-grade disease are diagnosed in the advanced stages, with over 70% of patients relapsing, and complete sustained responses to treatment are scarce [9].

Here, we describe a case of a heavily pretreated LGSOC patient, without a known BRAF-identified mutation, who had a complete response under treatment with trametinib. This response has been sustained for more than three years now.

## Case presentation

**Figure 1:**
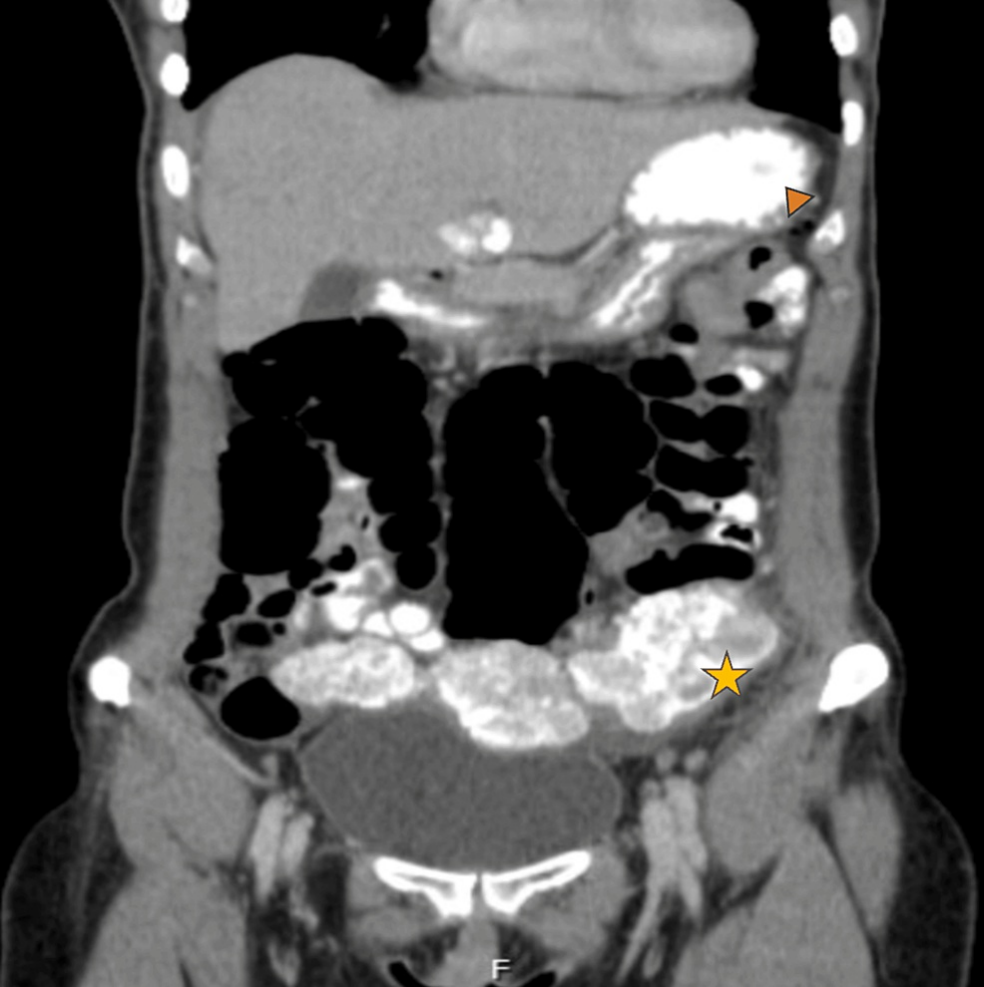
Imageological diagnosis of the pelvic mass. At diagnosis, the tumor presented as an unresectable pelvic mass (star) with extensive carcinomatosis with multiple splenic (arrowhead) and capsular implants. The biopsy confirmed the diagnosis of a low-grade serous adenocarcinoma.

In January 2013, a 45-year-old female (gravida 1, para 1), with an unremarkable previous medical history, presented with a two-month history of abdominal bloating and vomiting; she was originally diagnosed with extensive carcinomatosis with multiple splenic and capsular implants and a large unresectable pelvic mass (Figure 1). CA-125 was 420 U/mL at diagnosis. Biopsy was compatible with low-grade serous adenocarcinoma. Although surgery was discussed, the tumor was considered unresectable by radiological criteria, and it was decided to start neoadjuvant platin-based chemotherapy with carboplatin area under the curve (AUC) 5 and paclitaxel 175 mg/m^2^ (four cycles). The patient underwent intermediate cytoreduction - total abdominal hysterectomy, bilateral salpingo-oophorectomy, omentectomy, splenectomy, appendectomy, and tumor debulking, with only residual tumor found by CT scan, being a 14 mm psammomatous implant on the teres ligament. Pathology confirmed stage III-C low-grade serous ovarian carcinoma, with invasion to the ovarian capsule, uterine serous membrane, spleen, and all excised nodules, which included perihepatic and subdiaphragmatic nodules. Peritoneal cytology was positive for adenocarcinoma cells. After completing four more cycles of platin-based adjuvant chemotherapy, a clinical and biochemical complete response was observed (CA-125: 5.8 U/mL).

Twenty-nine months later, in March 2016, a supraclavicular node was confirmed to be metastatic, and a CT/PET scan disclosed an additional hepatic nodule (SUV 8.5). The patient’s CA-125 level increased to 41 U/mL. Additional imaging studies by MRI disclosed multiple nodular carcinomatosis. As the patient refused chemotherapy, she started hormonal therapy with tamoxifen (20 mg/day). Six months later, after clinical and biochemical progression (supraclavicular adenopathy and CA-125: 91.2 U/mL), she switched to the aromatase inhibitor (AI) letrozole (2.5 mg/day). Three months later, after disease progression (confirmed radiologically), platinum-based chemotherapy associated with bevacizumab was proposed, but the patient chose to delay chemotherapy, starting bevacizumab (10 mg/kg, every two weeks) while maintaining AI. Objective clinical, biochemical, and radiological stabilization was observed for over two years.

**Figure 2:**
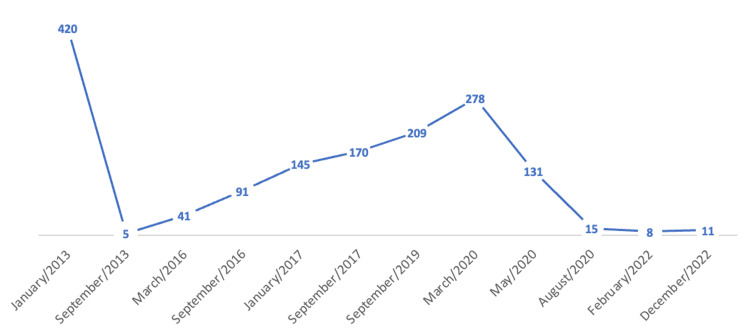
Evolution of the CA-125 marker.

After 28 months, grade 2 proteinuria was observed, as well as objective disease progression, characterized by higher metabolic activity of the previous peritoneal nodes (SUV 8.1, compared to previous 6.3, and SUV 8.6, compared to previous 3.9) and a new pancreatic lesion with 5 mm. Monotherapy with trametinib was then started on a daily dose of 2 mg per day, in March 2020. After three days of treatment, a facial pustular rash, grade 1 alopecia, as well as other skin lesions (grade 3 toxicity), led to an early dose reduction to 1.5 mg/day and dermatological treatment (topical Sébium Sensitive and oral Novophane), as well as oral doxycycline (100 mg/day, one week) and topical metronidazole (7.5 mg/day at night). A complete biochemical and radiological response was observed by CT/PET scan in December 2020, after nine months of treatment. Further CT/PET scans and CA-125 marker evolution confirmed sustained response, the last one after 30 months of treatment (Figure 2). A further dose reduction to 1 mg/day was necessary at 26 months of therapy, due to hematuria.

After 36 months of treatment with trametinib (31 cycles), the patient remains fully ambulatory, with an Eastern Cooperative Oncology Group performance status (ECOG PS) of 0, and the radiological report is assessed as a complete response.

## Discussion

Patients with metastatic LGSOC usually have reduced sensitivity to chemotherapy regimens, and successive treatments frequently show lower progression-free intervals. In contrast to high-grade tumors; however, LGSOC may derive more benefit from hormonal therapy [10], bevacizumab [11], and MEK inhibitors [12]. The case we reported here is remarkable since our patient did not derive benefit from hormonal therapy, but after a long period of disease stabilization with bevacizumab, an objective complete response was achieved with trametinib. While MEK inhibition exerts very clear cytostatic effects upon tumor cells, in many cases, this suppression of proliferation is not accompanied by cell death. This is a likely cause of trametinib's inefficiency in many preclinical and clinical tests. In our patient, good clinical response is maintained after more than 30 months of treatment. Although two dose reductions were required due to adverse events, the only significant side effect has been grade 1 skin toxicity, which was managed with topical treatment. No long-term toxicities have been observed thus far.

We do not have data regarding the KRAS/BRAF status of the tumor. This represents the biggest limitation of the present report. While it is possible that females with tumors with KRAS or BRAF mutations may have a better outcome [6,13,14], the results of the GOG0281 trial showed that the benefit with trametinib was observed independently of the presence of these mutations [15]. Indeed, trametinib is recommended as a new standard of care for females with relapsed or persistent LGSOC.

**Figure 3:**
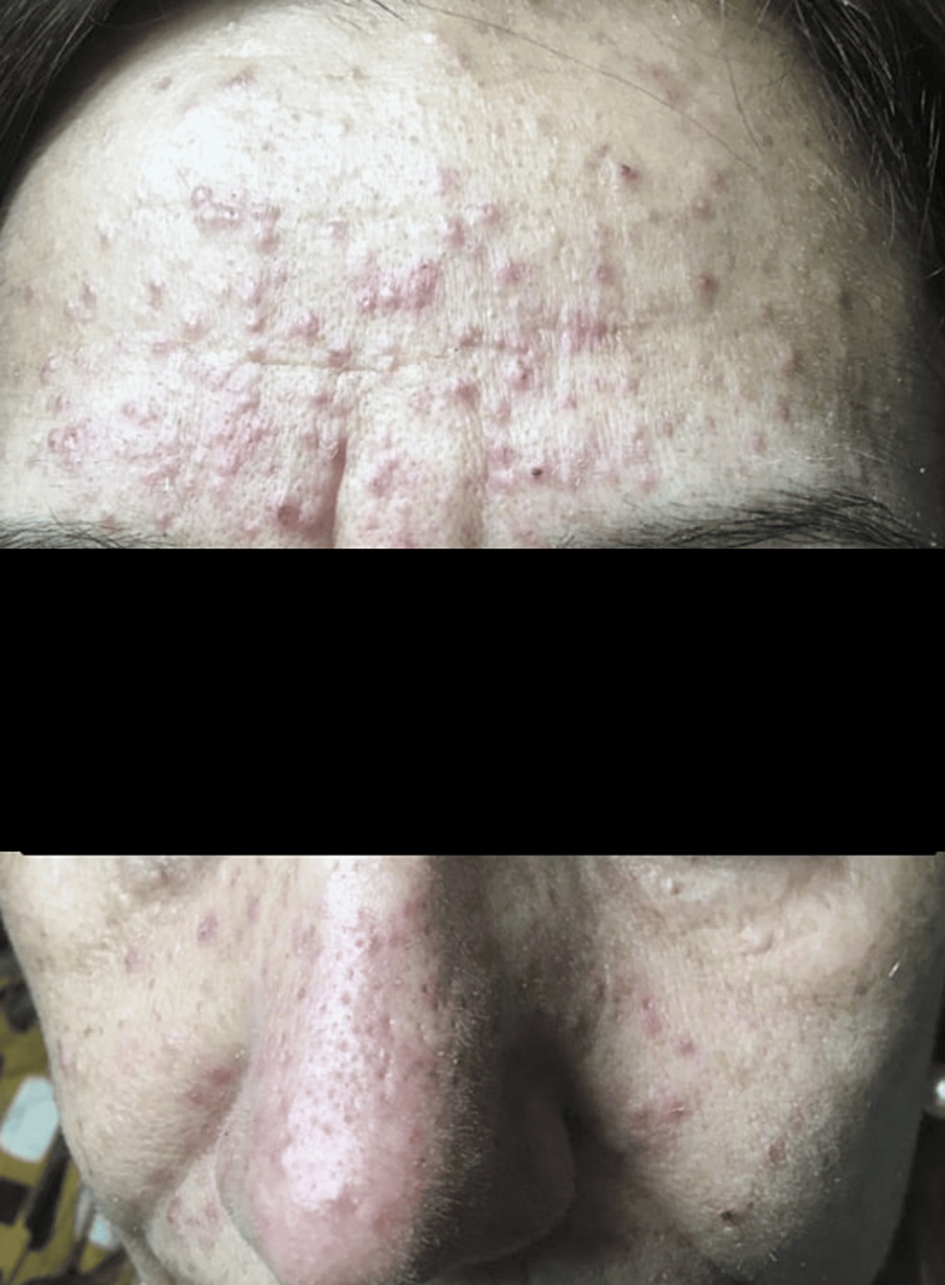
Facial acneiform rash (grade 3 toxicity) that started three days after the introduction of trametinib (2 mg/day). Treatment was suspended for one week, and the patient started on topical corticosteroids and urea-based cream, with complete resolution of the rash. This dermatological toxicity led to an early dose reduction to 1.5 mg/day without any further skin reaction.

In the GOG0281 trial, the median number of cycles received was eight, and 30% of patients required two dose reductions. As of March 2023, our patient continued to derive objective treatment benefits after 31 treatment cycles. Two dose reductions were necessary, the first due to skin toxicity (G3 acneiform rash), and the second because of hematuria (Figure 3). In comparison, one patient in the GOG0281 trial experienced a complete response, although details such as the BRAF mutation status of her tumor, previous therapy regimens, and duration of response are not available [15].

There is no established data on treatment duration in this setting, except in cases of disease progression, serious adverse events, or patient refusal. After discussing with the patient and ensuring ongoing safety surveillance, we have decided to continue the trametinib treatment.

To the best of our knowledge, this is the first reported case of a complete response to single-agent MEK inhibitor therapy in a patient with recurrent LGSOC, who has an unknown V600E BRAF mutation status, outside of the GOG0281 trial.

## Conclusions

Low-grade serous carcinoma of the ovary is characterized by MAPK pathway aberrations. Treatment with trametinib resulted in a complete response to a low-grade serous carcinoma of the ovary, after chemotherapy and hormonotherapy. The benefit of MEK inhibitors in these tumors may be independent of the presence of BRAF mutations.
